# A Neutrophil-Driven Inflammatory Signature Characterizes the Blood Transcriptome Fingerprint of Psoriasis

**DOI:** 10.3389/fimmu.2020.587946

**Published:** 2020-11-24

**Authors:** Arun Rawat, Darawan Rinchai, Mohammed Toufiq, Alexandra K. Marr, Tomoshige Kino, Mathieu Garand, Zohreh Tatari-Calderone, Basirudeen Syed Ahamed Kabeer, Navaneethakrishnan Krishnamoorthy, Davide Bedognetti, Mohammed Yousuf Karim, Konduru S. Sastry, Damien Chaussabel

**Affiliations:** ^1^ Research Department, SIDRA Medicine, Doha, Qatar; ^2^ Department of Internal Medicine and Medical Specialties, University of Genoa, Genoa, Italy; ^3^ Department of Pathology, SIDRA Medicine, Doha, Qatar

**Keywords:** psoriasis, transcriptomics, blood, Kawasaki disease, systems biology

## Abstract

Transcriptome profiling approaches have been widely used to investigate the mechanisms underlying psoriasis pathogenesis. Most researchers have measured changes in transcript abundance in skin biopsies; relatively few have examined transcriptome changes in the blood. Although less relevant to the study of psoriasis pathogenesis, blood transcriptome profiles can be readily compared across various diseases. Here, we used a pre-established set of 382 transcriptional modules as a common framework to compare changes in blood transcript abundance in two independent public psoriasis datasets. We then compared the resulting “transcriptional fingerprints” to those obtained for a reference set of 16 pathological or physiological states. The perturbations in blood transcript abundance in psoriasis were relatively subtle compared to the changes we observed in other autoimmune and auto-inflammatory diseases. However, we did observe a consistent pattern of changes for a set of modules associated with neutrophil activation and inflammation; interestingly, this pattern resembled that observed in patients with Kawasaki disease. This similarity between the blood-transcriptome signatures in psoriasis and Kawasaki disease suggests that the immune mechanisms driving their pathogenesis might be partially shared.

## Introduction

Inflammation has an important role to play as part of the host defense against infection. However, prolonged or excessive inflammation can cause notable pathology (1–3). One example of such a pathology is psoriasis, which affects ~100 million individuals worldwide (4). This common, immune-mediated disease results in a unique skin barrier abnormality caused by excessive epidermal proliferation and inflammation (5, 6). Psoriasis pathogenesis is likely driven by many factors, including environmental triggers, genetic susceptibility, and even microbiome composition (1, 6, 7). At the cellular level, an interaction between innate and adaptive immune responses, and the activation of Th17 and Th1 cells are key to the immunopathogenesis (8). Plasmacytoid dendritic cells (DCs) found in psoriatic skin are activated by antigens and subsequently release IFN-α. Meanwhile, myeloid DCs secrete IL-23 and IL-12, which favors T-cell differentiation into Th17 and Th1 pathways, respectively (9, 10). In turn, Th17-derived cytokines including IL-17A and IL-22 play a dominant role in driving keratinocyte activation and proliferation. Finally, TNF-α secreted by DCs, Th17, and Th1 cells, and keratinocytes in psoriatic skin amplifies and perpetuates inflammation. Current treatment modalities include topical glucocorticoids, vitamin D analogues, phototherapy, conventional immunosuppressives (e.g., ciclosporin, methotrexate), and various biologics that target TNF-α (e.g., infliximab), the IL-17 pathway (e.g., secukinumab), and IL12/IL-23 (e.g., ustekinumab) (9–12). But despite such progress in understanding the molecular mechanisms driving psoriasis, we are still far from having a complete understanding of the immunopathogenesis and developing highly effective therapeutics.

To gain a better understanding of psoriasis pathophysiology, many researchers have compared the transcriptome profiles of diseased *vs.* healthy skin tissues isolated from affected patients (13–17). By contrast, only a few research groups have profiled genome-wide transcript abundance in blood samples from patients (18, 19). Measuring transcript abundance in the blood might seem less applicable to studies of skin diseases such as psoriasis; however, the blood presents the advantage of being highly accessible and amenable to serial sampling. Thus, blood transcriptional profiling could be harnessed to monitor dynamic treatment responses. Another advantage is the availability of numerous public blood transcriptome datasets, which allows us to make comparative analyses across various inflammatory diseases.

Here, we compared the blood transcriptome fingerprints of two publicly available psoriasis datasets (18, 19) with those derived from a collection of 16 reference patient cohort datasets (20). Our functional interpretations relied on extensive annotations and expression patterns observed in purified leukocyte populations.

## Methods

### Collection of Public Datasets

The public datasets used in this re-analysis and interpretation were available in the NCBI GEO repository (21) (**Supplementary Table 1**). They include psoriasis blood transcriptome datasets as well as a collection of reference transcriptome datasets used for contextual interpretation. They are described in brief here:

Two psoriasis blood transcriptome datasets were identified for which data analysis was performed as detailed below. Both comprised control groups of subjects. Findings were reported in the literature by their original contributors:

- The GSE55201 dataset contributed by Wang et al. was generated using an Affymetrix U133 Plus (microarray) and consists of profiles for 81 samples. The study examined the role of IL-17 in ameliorating systemic inflammation and its impact on psoriasis complications, such as atherosclerosis and ischemic cardiovascular disease (19).
- The GSE123786 dataset contributed by Catapano et al. was generated using an Illumina HiSeq 2000 platform (RNA-Seq) and consists of profiles for 16 samples. This study examined the involvement of IL-36 in extracutaneous manifestations of psoriasis (18).

Other blood transcriptome datasets were used as reference:

- The GSE100150 dataset (20) contributed by our group was generated using an Illumina HumanWG-6 v3.0 BeadChip and consists of 16 reference patient cohorts encompassing the profiles of 985 subjects/samples.

Two other reference datasets were used to functionally interpret gene signatures:

- The GSE24759 dataset contributed by Novershtern et al. (22) was generated using Affymetrix U133A GeneChip and consists of 211 samples. The samples were collected from 4 to 7 donors and a wide range of hematopoietic cell populations from both adult and cord blood were profiled.
- The GSE60424 dataset (23) contributed by our group was generated using an Illumina RNA-Seq platform and consists of 134 subject/samples profiles. In this study, leukocyte populations were isolated from the blood of healthy individuals and patients with diabetes mellitus type 1, amyotrophic lateral sclerosis, multiple sclerosis (MS; pre- and post-interferon treatment) or sepsis.

### Data Processing

The analysis workflow determines for each module the proportion of its constitutive transcripts that significantly differ in comparison with a given baseline (e.g., healthy controls). Thus, at the module level, changes are expressed as the proportion of transcripts constituting a given module being significantly increased (0 to 100%) or decreased (0 to −100%) compared with healthy controls. By design, changes in abundance among transcripts within a given module tend to be coordinated. However, when both significant increases and decreases are observed for the same module, the dominant trend is retained.

Data pre-processing steps for the two public psoriasis blood transcriptome datasets were performed as follows: The Wang *et al.* dataset (GSE55201) was generated using Affymetrix GeneChip and normalized with GCRMA (24). The Catapano *et al.* dataset (GSE123786) was generated *via* RNA-Seq data and the data are presented as RPKM values (reads per kilobase of transcript, per million mapped reads) after mapping with the HG38 genome build; the read counts were calculated using htseq-count (25).

### Transcriptional Module Repertoire Analyses

Modular repertoire analyses were performed at the group level on both GSE55201 and GSE123786 psoriasis datasets using the BloodGen3Module R package: https://github.com/Drinchai/BloodGen3Module (26). The pre-determined repertoire of 382 co-expressed blood transcriptional modules that served as a framework for this analysis was described by Altman et al. (20). Briefly, it was constituted based on co-expression observed across a collection of 16 reference datasets encompassing 985 unique blood transcriptome profiles and 14,168 transcripts. A wide range of immune states are represented in this collection of reference datasets, including several infectious diseases, autoimmune diseases, inflammatory disorders as well as cancer, pregnancy, and solid organ transplantation. Because this module repertoire is “fixed” and destined for reuse as a generic framework for blood transcriptome analyses, considerable efforts were dedicated to its annotation and functional characterization. This work included functional enrichment analyses (ontologies, pathways, literature terms), and the generation of heatmaps representing transcript abundance patterns for reference datasets. The latter included, for instance, profiling data from isolated leukocyte populations. Interactive presentations were established to provide access to the large compendium of analysis reports and heatmaps that were generated as part of these annotation efforts. The presentation for a subset of 21 modules associated with inflammation that will be discussed in more detail as part of this work can be accessed *via* this link: https://prezi.com/view/GkH4wHb0jhIbDGt7Ibwi/. A demonstration video can be accessed *via* this link: https://youtu.be/fTfqGhcCNdE. However, it should be noted that module annotation has an element of subjectivity and is still a work-in-progress. Additionally, some of the functional “labels” that have been assigned are still tentative and subject to change as data analyses and interpretations progress across several projects.

### Blood Transcriptome Fingerprint Visualizations

The percent of increased or decreased transcripts computed per module were represented on a fingerprint grid plot. In brief, modules occupy a fixed position on the grid and changes for that module are indicated by a red spot (increased abundance compared to controls) or a blue spot (decreased abundance compared to controls). All fingerprints plots show changes in transcript abundance in cases compared to respective healthy controls (run concomitantly and matched for demographics). Modules arranged on the same row belong to one of 38 “module aggregates.” These aggregates are formed based on similarities in the patterns of transcript abundance changes across the 16 reference datasets. Thus, a vertical reading of the grid across the rows gives an indication of the patterns of change in a given set of patients at the least granular level (aggregates). A second horizontal reading within each row and across the columns gives an indication of the changes occurring at a more granular level (modules). Functional interpretations are indicated by a color code that is overlaid on the grid plot (**Supplementary Figure 1**). Because the positions of the modules on the grid are fixed, different fingerprints generated for independent groups of patients can be compared. Fingerprint grid plots for all of the 16 reference cohorts can be generated dynamically using a previously developed app: https://drinchai.shinyapps.io/dc_gen3_module_analysis/#.

### Screening of Drug Targets

Transcripts among the A35 modules were screened for the presence of drug targets using the “open targets platform” that is available *via* the open targets consortium at https://www.targetvalidation.org/ (27). The batch query functionality was used. The transcripts encoding targets of existing drugs (referred to in the results as “targets with clinical precedence”) were retrieved (**Table 1**).

**Table 1:** Transcripts comprised in aggregate A35 for which the gene products are targetable by existing drugs, and the drugs tested in psoriasis or Kawasaki disease (see *Methods*).

ID	Drug targets with clinical precedence	Drugs tested with psoriasis as an indication and their corresponding targets (underlined)	Drugs tested with KD as an indication	Open targets report
M15.84	MAPK14	MAPK14: BMS-582949 (phase II), doramapimod (phase II)	None	https://bit.ly/3iynGZF
M13.16	CASP4, CASP5, CSF2RB, CXCR2, KCNJ2, MGAM, NAMPT	CXCR2: navarixin (phase II)	None	https://bit.ly/38x3sey
M13.1	CASP1, FGR, FKBP1A, IMPDH1, MAPK1, MAPK3, S1PR4, NCSTN, NOTCH1, PRKCD, RARA, RXRA, SELL, SYK, TNFSF13B	RARA/RXRA: acitretin (phase IV), tazarotene (phase IV), etretinate (phase IV), alitretinoin (phase II); FKBP1A: tacrolimus (phase III); SELL: bimosiamose (phase II); S1PR4: amiselimod (phase II), PRKCD: sotrastaurin (phase II); IMPDH1: mycophenolate mofetil (phase II).	None	https://bit.ly/3iD3CW7 https://bit.ly/3e5UqGp https://bit.ly/3e3XQJF https://bit.ly/2W76iSz https://bit.ly/38uAT18 https://bit.ly/3e17rkn
M15.37	IL1B, NDUFB3,	NDUFB3: metformin (phase III)	None	https://bit.ly/3iBFByI
M15.113	BMX, IL1R1, MAPK14	None	None	
M12.10	ALOX5, IL13RA1, RAF1, TBXAS1, TNFRSF1A	None	None	
M13.12	CA4, F5, FCGR1A, HPSE, MMP9, TLR5	None	None	
M15.105	PSMB3	None	None	
M15.109	IL6R, NAMPT	None	None	
M13.22	C5AR1, FGR, HCK, HSPA1A, IFNAR1, IL8RA (CXCR1), LY96	IL8RA (CXCR1): Navarixin (Phase II)	None	https://bit.ly/2ZLoOAV
M14.28	None	None	None	
M15.26	EGLN1, FKBP1A, HPSE, MCL1, TLR4	FKBP1A: tacrolimus (phase III)	None	https://Bit.ly/3e5UqGp
M14.65	CD14, IFNGR2, ITGB2	IFNGR2: interferon gamma-1b (phase 0, terminated)—actimmune (early phase I)	None	https://bit.ly/3f77kVR
M16.79	CASP4, IL10RB	None	None	
M16.98	ADORA2B, CACNA1E, VDR	ADORA2B: caffeine (phase I completed) VDR: calcipotriene (phase IV), calcitriol (phase II), becocalcidiol (phase II), pefcalcitol (phase II), ergocalciferol (psoriasis vulgaris-phase 0), cholecalciferol (phase 0)	None	https://bit.ly/38wYAGh
M13.3	APH1B, CSF2RA, GBA, HDAC4, MAPK1, OPRL1, PDK3, PIM3	None	None	
M14.7	ECGF1, JAK2	JAK2: tofacitinib (phase III), baricitinib (phase II), ruxolitinib (phase II), lestaurtinib (phase II), peficitinib (phase II)	None	https://bit.ly/3f6RlHf
M14.74	None	None	None	
M15.43	COL18A1, MGC18216 (IGF1R), PTPRC, TNFSF14, TXNRD1	None	None	
M15.78	ANPEP, CSF3R, IL4R	None	None	
M15.81	PIK3CD	None	None	

## Results

### Blood Transcriptome Signatures of Independent Psoriasis Datasets Share a Similar Modular Component

We first aimed to determine whether we could measure robust changes in transcript abundance in the blood of psoriasis patients in comparison to heathy controls. We presumed that such signatures, if present, could then be “benchmarked” against that of other inflammatory or autoimmune diseases.

**Figure 1:**
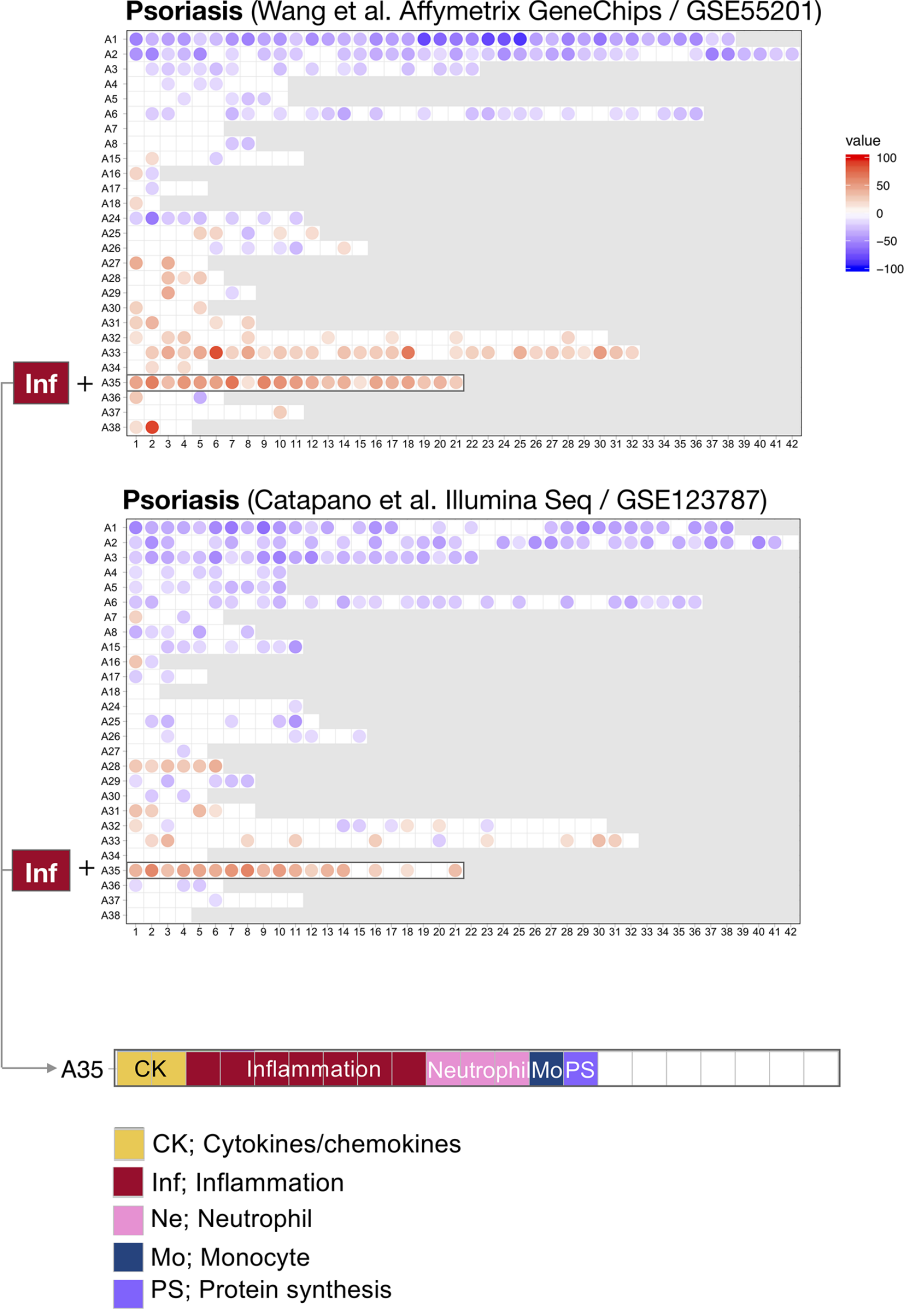
Blood transcriptome fingerprints of psoriasis. Differences in the levels of blood transcript abundance in patients with psoriasis and controls were mapped on a grid for the two public datasets from Wang *et al.* (GSE55201: 30 controls and 51 subjects) and Catapano *et al.* (GSE123786: 7 controls and 9 subjects). Each position on the grid is occupied by a given module (pre-determined set of co-expressed genes). A blue spot indicates a module for which constitutive transcripts are predominantly present at lower levels in patients *vs.* controls. Conversely, a red spot indicates a module for which constitutive transcripts are predominantly present at higher levels in patients *vs.* controls. No spots on a white background indicate that there are no changes for the module in question. A gray background means that there are no changes in the module at this position. The modules are arranged by rows in “module aggregates” and ordered by their similarity in expression patterns across a set of 16 disease or physiological states (reference dataset collection). A consistent increase was observed for modules constituting aggregate A35. This aggregate is highlighted on the grid and functional annotations are provided (bottom panel). Functional annotations for the entire gird are provided in **Supplementary Figure 1**.

For this first step, we harnessed data from two psoriasis blood transcriptome datasets of a relatively modest size that have been published and made available *via* the NCBI GEO repository (18, 19). The technology platforms used to generate each dataset were quite dissimilar: Wang *et al.* used microarrays while Catapano *et al.* performed RNA sequencing. We previously showed that differences in transcript abundance summarized at the level of coordinately expressed gene sets (modules) are more amenable to cross-platform comparisons than when differences are expressed at the individual gene level (28). We therefore used a pre-determined repertoire of blood transcriptome modules that was recently developed and characterized by our group (20) (see *Methods*). Briefly, we formed this repertoire on the basis of co-expression measured across 16 reference patient cohorts, encompassing 985 unique blood transcriptome profiles. Two-dimensional reduction levels are built into the repertoire. The least reduced level has 382 variables, which are the modules that are constituted by sets of genes. The most reduced level has 38 variables, which are module aggregates that are constituted by sets of modules that altogether encompass the 382-module repertoire. Changes between cases and controls are expressed as a proportion of the transcripts constituting a given module found to be significantly increased (max +100%, all transcripts are increased) or decreased (−100%). We thus determined differences in transcript abundance for each of the 382 modules for the Wang *et al.* and Catapano *et al.* datasets. We represented these differences on a fingerprint grid plot, where the assignment of modules to a given position on the grid was fixed (**Figure 1**).

The fact that positions of modules on the grid are fixed ensures that the generated fingerprints are directly comparable. Here, we found a good level of concordance between the two datasets, with both predominantly showing changes for the 21 modules forming row A35 on the grid. At a high level, the module aggregate A35 is functionally associated inflammation (detailed below). In addition, the Catapano *et al.* dataset showed increases for modules forming row A28. The module aggregate A28 is functionally associated with interferon responses. Notably, an interferon signature was also reported by Catapano and colleagues (18), and seems to be associated with generalized pustular psoriasis, which is a severe form of the disease (1).

**Figure 2:**
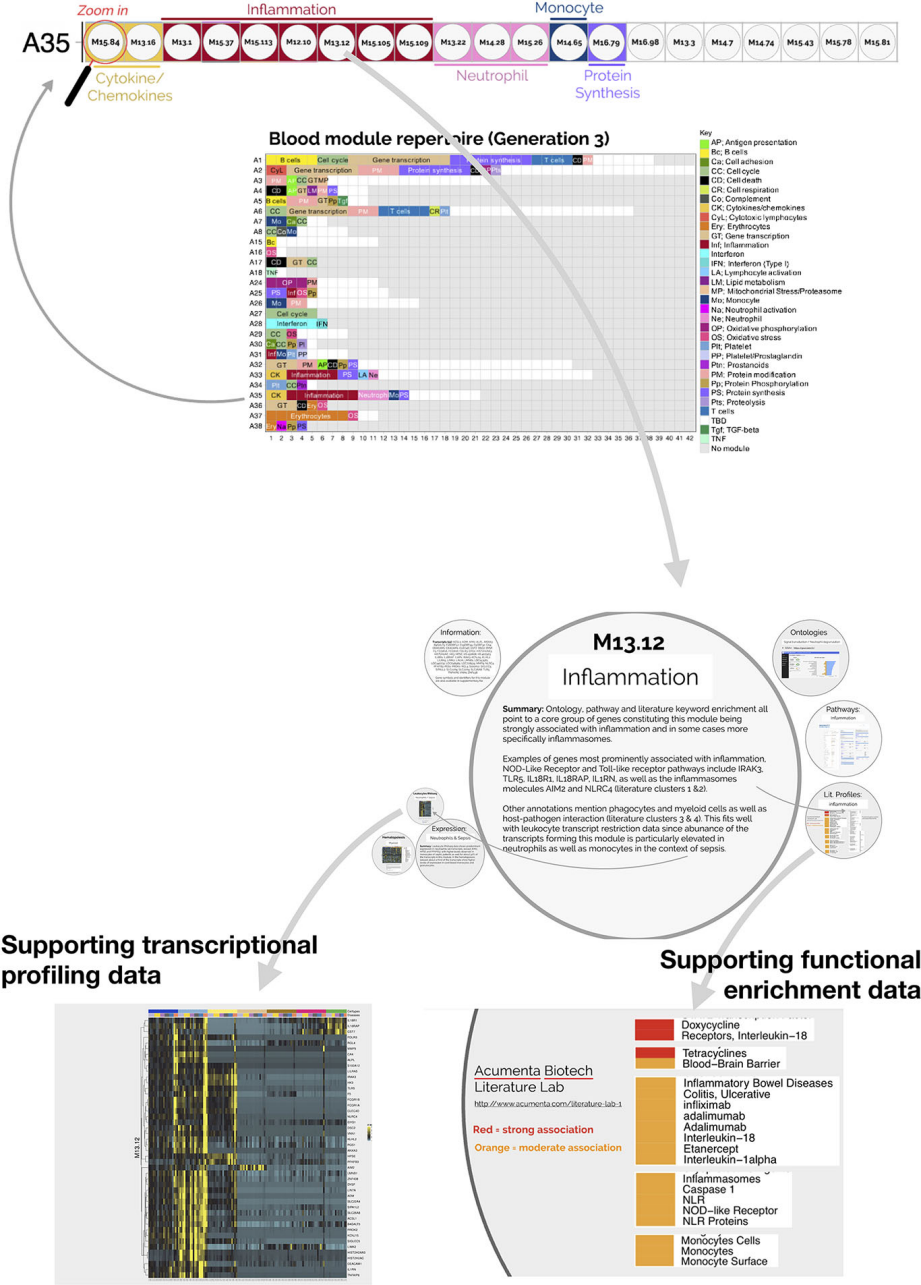
Interactive presentation providing transcriptional profiling and functional enrichment data for modules constituting aggregate A35. An interactive presentation has been developed that allows for exploration of the modules constituting aggregate A35. A gene list is provided for each module, along with gene ontology, pathway or literature term enrichment results and transcriptional profiling data for the reference transcriptome datasets (circulating leukocyte populations, hematopoiesis). A summary of the findings is also given. The interactive presentation is available *via*: https://prezi.com/view/7Q20FyW6Hrs5NjMaTUyW/. The presentation provides zoom in/out functionalities for close-up examination of the text and figures embedded in the presentation.

The fact that an increase in abundance of A35 modules was observed in both datasets suggests that this modular signature constitutes the main component of the blood transcriptome fingerprint associated with psoriasis overall. At a high level, seven of the 21 modules forming aggregate A35 were associated with inflammation, three with neutrophils, two with cytokines/chemokines, one with macrophages, and one with protein synthesis (**Figure 1**). The remaining seven modules were not associated with any given functional annotations due to lack of convergence between the functional profiling results obtained *via* different methodologies. The reports from gene ontology (GO), pathway and literature keyword enrichment analyses upon which these determinations were made (**Figure 2**), are available *via* an interactive presentation (https://prezi.com/view/7Q20FyW6Hrs5NjMaTUyW/) and all functional annotations of the A35 module are readily available (**Table 2** and **Supplementary File 1**).

**Table 2:** The 21 modules constituting aggregate A35.

ID	Grid position	Number of transcripts	Functional annotation	Representative genes
M15.84	A35-1	20	Cytokines/chemokines	S100P, TLR2, MAPK14, FCAR
M13.16	A35-2	39	Cytokines/chemokines	BTNL8, CR1, FFAR2, FPR2, TLR6, ALPK1
M13.1	A35-3	137	Inflammation (innate immune response activation)	PYCARD, CLEC4A, SYK, CD300A, PRKCD, PELI1, LILRA2, MYD88, HSPA1B
M15.37	A35-4	33	Inflammation (leukocyte migration)	LAT2, SLC7A8, IL1B, FPR2, SLC16A3, GPSM3
M15.113	A35-5	16	Inflammation	SOCS3, RAB20, MAPK14, BMX, RASGRP4
M12.10	A35-6	53	Inflammation (neutrophil degranulation)	CRISPLD2, ALOX5, LAMP2, RAB24, ITGAX, TIMP2, SIRPA, RNASE3, LILRB3, IGF2R
M13.12	A35-7	55	Innate immunity, myeloid cells, inflammasomes	AIM2, TLR5, SIGLEC5, IL18RAP, IL18R1, S100A12, NLRC4, IRAK3, TNFAIP6, CLEC4D, LILRA5, FCGR1A, FCGR1B
M15.105	A35-8	16	Inflammation (myeloid cells, arginase pathway)	MAP3K3, TYROBP, PSMB3, LILRB2
M15.109	A35-9	17	Inflammation (defense response, leukocyte migration)	IL1RN, IL6R, TNFRSF10B, CR1, TLR8, FCGR2A
M13.22	A35-10	65	Neutrophils (response to LPS)	AKIRIN2, SLC11A1, C5AR1, LY96, TRIB1, LITAF, IFNAR1
M14.28	A35-11	20	Neutrophils (neutrophil degranulation)	BST1, MMP25, SERPINA1, FCER1G, ITGAM, SLC2A3, LILRA2, OSCAR
M15.26	A35-12	38	Neutrophils (activation, exocytosis)	PREX1, CEACAM3, ATP8B4, PLAUR, RAB27A, HPSE, SIRPB1
M14.65	A35-13	15	Monocyte (host defense)	ITGB2, CYBA, CD14, GNS, RAB7A, IFNGR2
M16.79	A35-14	27	Protein synthesis (secretion)	PYCARD, CNN2, FAM49B, RHOT1, DNAJC5, GAPDH, MCU, LILRA5
M16.98	A35-15	18	TBD	IL22, VDR, KREMEN1, LOXL3, ADORA2B, MAK, TIFA
M13.3	A35-16	100	TBD (response to stress)?	ERO1A, MAP3K2, G6PD, GADD45A, EDEM2, GBA, WIPI1
M14.7	A35-17	31	TBD	MFN2, JAK2, BATF, TFE3, CPEB3
M14.74	A35-18	14	TBD	MOSPD2, CD58, CKLF, CD53, TLE4, RNASEL
M15.43	A35-19	30	TBD (protein secretion)?	RCN3, COP1, CARD16, CLEC4E, CAMK2G
M15.78	A35-20	20	TBD (signal transduction)?	CSF3R, IL4R, SEMA4B, MKNK1, CREBRF, GPAT3, REM2
M15.81	A35-21	20	TBD (neutrophil degranulation)	PKM, GAA, ALDOA, AGPAT2

Detailed information can be found in **Supplementary File 1**, and an interactive presentation that is accessible via this link: (https://prezi.com/view/GkH4wHb0jhIbDGt7Ibwi/; demonstration video (https://youtu.be/0j-kcE1tlXAc). (Acronym TBD, to be determined).

In summary, this step identified that modules forming aggregate A35 are conserved between two independent psoriasis blood transcriptome datasets. Notably, this convergence was evident even though distinct technology platforms were used to generate the respective datasets. Altogether, these findings indicate that a blood transcriptional signature can consistently be observed in the blood of psoriasis patients.

### The Psoriasis Blood Transcriptome Signature Is Associated With Neutrophils and Inflammation

We next aimed to determine the relevance of the increase in A35 transcripts in the context of psoriasis pathogenesis. To do so, we proceeded with the functional interpretations of this signature.

The two converging themes that emerged through the extensive annotation work mentioned above were “neutrophil” and “inflammation.” For instance, enriched literature terms included “neutrophil degranulation,” “inflammation,” and “inflammasome.” Consistently, some of the genes in these modules are most recognizable as being involved in inflammatory processes, including those coding for inflammasome components. For example, NLR protein families were found across different modules within this aggregate, including NLRX1, NLRC4, NLRP12. Furthermore, “neutrophil activation involved in immune responses” (GO:0002283) was one of the most over-represented GO terms, with 121/784 transcripts forming modules belonging to aggregate A35. Thus, both gene composition and functional enrichment analyses suggest that this set of 21 modules constituting aggregate A35 is involved in inflammatory processes.

**Figure 3:**
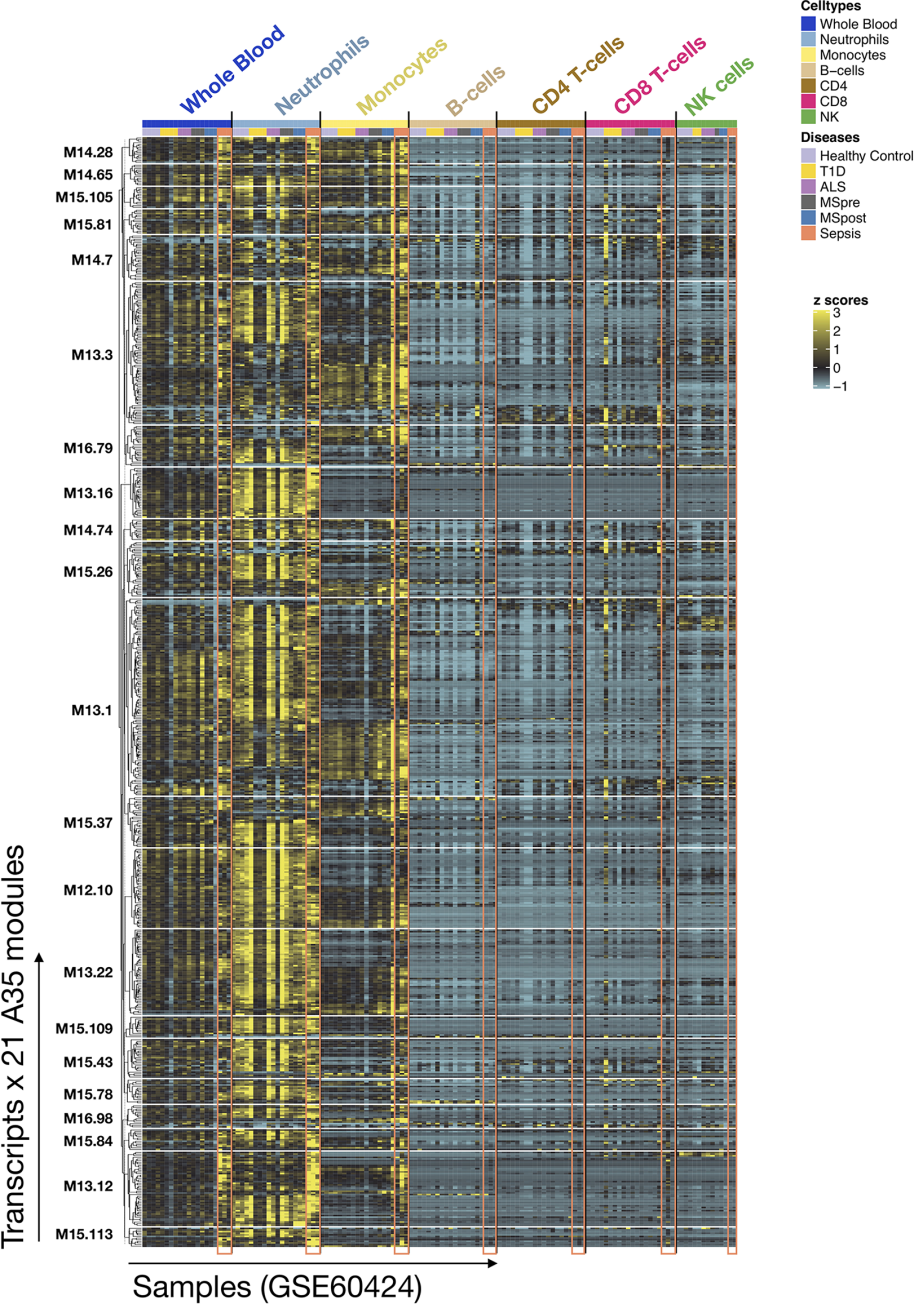
Expression patterns of genes constituting A35 modules across whole blood and purified leukocyte populations. The expression levels for genes constituting A35 modules are shown on a heatmap for a reference dataset comprising the profiles of isolated leukocyte populations (GSE60424). The rows represent genes, with each cluster of rows representing a module. The columns represent samples. This study compared the whole transcriptome signatures of six immune-cell subsets and whole blood from patients with one of an array of immune-associated diseases. Fresh blood samples were collected from healthy subjects and those diagnosed with type 1 diabetes, amyotrophic lateral sclerosis, sepsis, or multiple sclerosis (before and 24 h after the first dose of IFN-beta). RNA was extracted from each of the indicated cell subsets and whole blood samples, and then processed for RNA sequencing (Illumina TruSeq; 20M reads).

To complement our functional profiling analyses, we examined the expression patterns of the genes belonging to module A35 in reference transcriptome datasets (see *Methods*). In particular, a dataset that we previously generated and deposited in the GEO showed that among circulating leukocyte populations, the expression of A35 transcripts was predominant in neutrophils [Linsley et al. (GSE60424) (23), http://sepsis.gxbsidra.org/dm3/miniURL/view/Q0)] (**Figure 3**). In another dataset, also contributed by our group, we found that the expression of A35 transcripts was upregulated in neutrophils exposed to plasma from septic patients *in vitro* (29); http://sepsis.gxbsidra.org/dm3/miniURL/view/Q2.

Altogether, functional and gene expression profiles observed in this reference dataset suggest that the A35 signature is associated with neutrophil-driven inflammation.

### The Blood Transcriptome Fingerprint of Psoriasis Resembles That of Patients With Kawasaki Disease

As mentioned, a benefit of examining transcriptome signatures in blood rather than skin samples from patients with psoriasis is that it lends itself to making comparisons across a wide range of diseases. Carrying out such comparisons allows us to draw parallels or identify differences with diseases for which the pathogenesis might be better understood and managed clinically.

**Figure 4:**
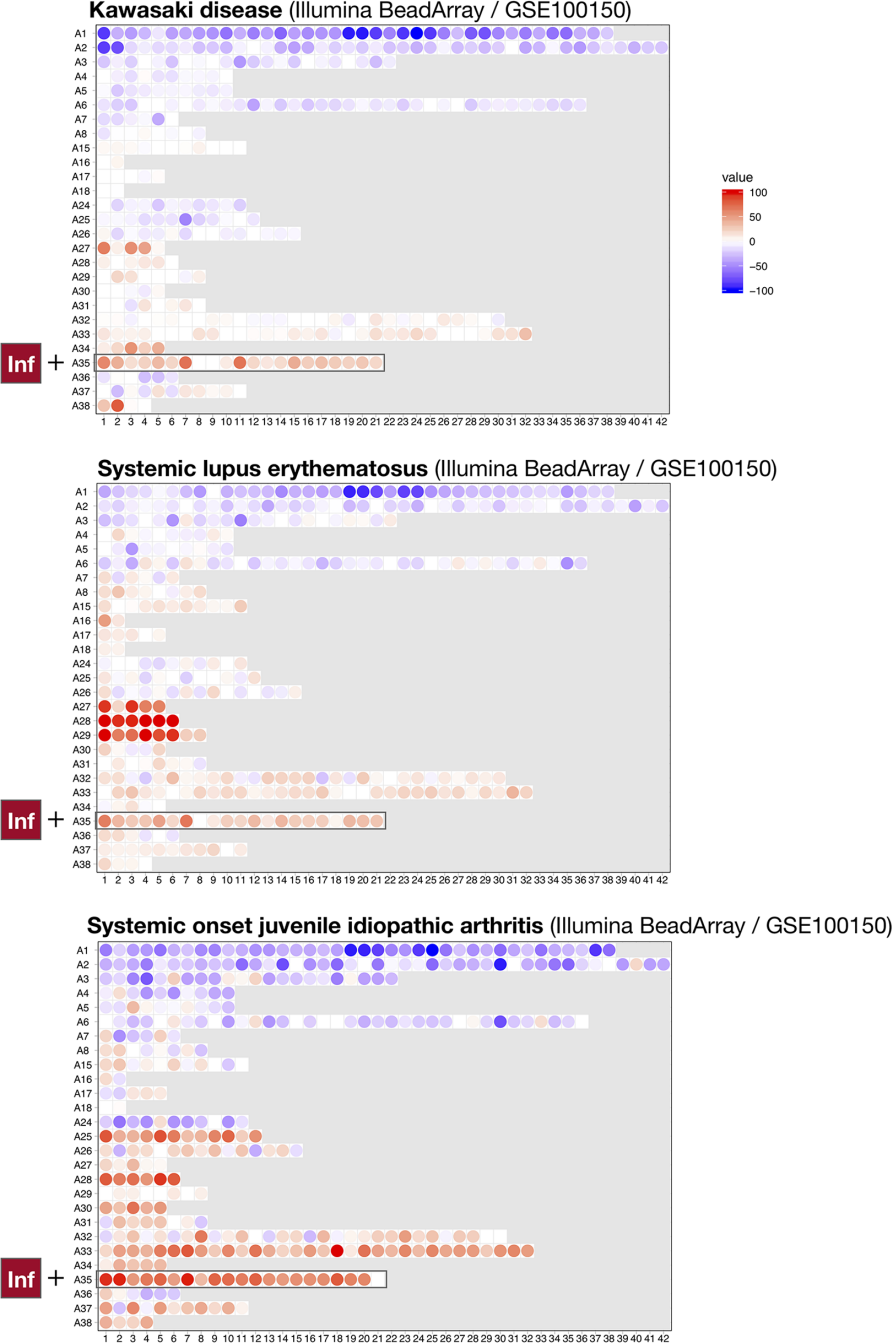
Blood transcriptional fingerprints of other autoimmune or autoinflammatory diseases. The differences in the levels of transcript abundance in the blood of patients with Kawasaki disease (top), systemic lupus erythematous (middle), or systemic onset juvenile idiopathic arthritis (bottom) are mapped on a grid, as described in **Figure 1**. The modules belonging to aggregate A35 are highlighted on this grid.

**Figure 5:**
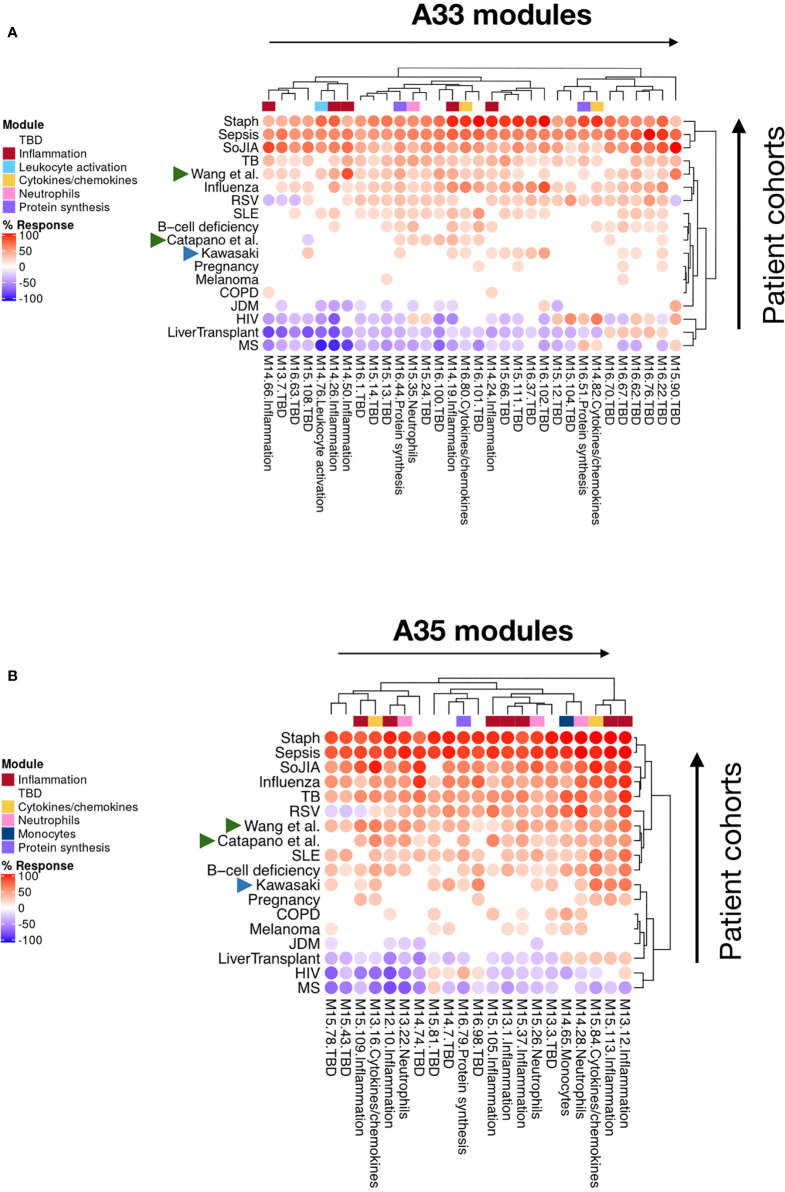
Patterns of abundance for modules forming aggregates A33 **(A)** and A35 **(B)**. Heatmaps displaying the changes in transcript abundance for modules (columns) belonging to two aggregates associated with inflammation (A33 and A35), across 16 reference datasets and two psoriasis datasets (rows). The two psoriasis datasets are designated by the names of the researchers who contributed them and are indicated by green arrowheads. An increase or decrease in the abundance of transcripts constituting these modules is shown by a red or blue spot, respectively. The rows (datasets from each disease cohort) and columns (modules) were arranged by hierarchical clustering based on similarities in patterns of transcript abundance.

To achieve this, we compared the module repertoire fingerprints of psoriasis with those of the 16 other diseases comprising the reference collection of datasets used to construct our module repertoire (20). As was the case for psoriasis, we observed an increase in abundance of A35 modules in the blood repertoire fingerprints of systemic lupus erythematous (SLE), systemic onset juvenile idiopathic arthritis (SoJIA), and Kawasaki disease (**Figure 4**). In the case of SLE and SoJIA, the increase in abundance of A35 transcripts was one of many “perturbations” of the blood transcriptome repertoire, which is consistent with the systemic inflammation that characterizes these two diseases [for example: modules in aggregates/rows A27-A29 (SLE) or A30-A38 (SoJIA)]. The fingerprints of patients with acute infections (e.g., bacterial sepsis, tuberculosis, or influenza infection) also showed pronounced changes (such fingerprints can be generated dynamically *via* our web application accessible at: https://drinchai.shinyapps.io/dc_gen3_module_analysis/#). Notably, the fingerprints of patients with sepsis closely resembled those of patients with SoJIA. In both pathologies, other modules functionally associated with inflammation, such as A33, also showed a robust increase in abundance; such increases were not observed in the context of psoriasis or Kawasaki disease. Indeed, patterns of abundance of A33 and A35 modules across the 16 reference patient cohorts and two psoriasis datasets indicated that A35 modules tend to be more ubiquitously increased in comparison to A33 modules (**Figure 5**). The relative difference in intensity of A33 and A35 signatures between the two psoriasis datasets also suggests that those signatures might be non-synonymous and represent distinct inflammation pathways. Conversely, the Kawasaki disease blood transcriptome repertoire fingerprint was more subtle and, like that of psoriasis, was mostly restricted to an increase in abundance of A35 module transcripts (**Figure 4**). This finding might reflect the fact that inflammation is typically localized at the onset of these diseases: to the skin for psoriasis and the vasculature endothelium for Kawasaki disease.

Taken together, these results suggest that the extent of changes in blood transcript abundance tends to correlate with the disease manifestation, from local (a low number of modules perturbed) to generalized inflammation (a high number of modules perturbed). Furthermore, the A35 transcriptional modules constituted the least common denominator across these inflammatory pathologies as it was the only set for which increases in transcript abundance were observed in all these immune-mediated diseases.

### A35 Modules Comprise Transcripts Which Are Targetable by Existing Drugs

The identification of a robust transcriptional signature in the blood of psoriasis patients has several implications. For instance, it opens up the possibility of including blood transcript profiling assays in patient monitoring studies. For instance, such studies might be designed to predict the risk of flares or to monitor responses to therapy. We thus went on to examine the presence of transcripts among A35 modules that encode molecules that are targets for existing drugs and could be included in immune monitoring panels.

Among the 784 transcripts constituting the A35 modules, 81 are encoding targets for existing drugs (see *Methods*) (**Table 1**). Among these, we sought to identify targets for which drugs have been tested in clinical trials for psoriasis (14 targets) or Kawasaki disease (none). Notable examples for targets among A35 transcripts for which drugs have been considered for treatment of psoriasis include JAK2. This member of the Janus kinase family participates in signaling events downstream of a broad range of cytokine and hormone receptors. Drugs targeting this molecule that have been tested in the context of psoriasis include tofacitinib, which seems to be safe and to confer a clinical benefit (30, 31). Other immunosuppressive drugs have also been evaluated, such as the selective, pan-protein kinase C inhibitor sotrastaurin that inhibits the kinase PRKCD (32, 33).

Overall, we found that a sizeable number of transcripts comprised in A35 modules encode targets for existing drugs: a minority of these have already been tested in patients with psoriasis. We posit that other suitable candidates might be included in this list that have not yet been evaluated in the context of psoriasis. Furthermore, given the parallels in the blood transcriptome signatures of psoriasis and Kawasaki disease, there is good cause to consider investigating repurposing drugs showing clinical benefit in patients with psoriasis for the treatment of patients with Kawasaki disease.

## Discussion

Involved skin tissue is the ideal sample source to investigate psoriasis pathogenesis. However, despite being less relevant to this disease, blood presents the advantage of being amenable to repetitive sampling with minimal risk or discomfort. The blood can also harbor information regarding the immune status of affected patients. Such information can be obtained *via* blood transcriptome profiling, whereby all RNA species that are present in a given sample are measured simultaneously. Maybe more importantly, vast amounts of blood transcriptome profiling data are available in public repositories that can be used for contextual interpretation and “benchmarking” of blood transcriptional signatures.

Here, we compared the blood transcriptome fingerprints derived from several inflammatory diseases with those derived from patients with psoriasis. We found that blood transcriptome profiling may indeed serve to assess the extent of systemic involvement in these pathologies. Interestingly, we saw that the repertoire of changes characterizing the psoriasis blood transcriptome signature is much narrower than what is observed in other systemic inflammatory diseases. We also established that modules assigned to the aggregate A35 and associated with neutrophil-driven inflammation are a hallmark of the psoriasis blood transcriptome signature.

The role played by neutrophils in psoriasis pathogenesis has received particular attention over recent years (34, 35). Consistently, the data from our study suggest that blood transcriptome profiling studies might be of value for further patient-based investigations. While such an approach has been relatively under-utilized in this context, our findings suggest the possibility of employing blood transcriptional profiling as a means to assess the extent of systemic inflammation in psoriasis patients. Whether these measurements add value to those obtained using more traditional inflammatory markers (e.g., measurement of serum protein markers or neutrophil: lymphocyte ratios) remains to be investigated. It may be particularly relevant to assess utility of such blood transcriptional markers for the evaluation of cardiovascular diseases (CVD) risk in patients suffering from inflammatory disorders. Indeed, systemic inflammation associated with psoriasis was recently linked with development of CVD in this patient population (36, 37), while the risk of cardiovascular symptoms in Kawasaki disease is well established (38). And the question of the relative benefits of the available psoriasis treatment options with regards to addressing this risk remains to be fully addressed (36, 39).

Blood transcriptome profiling may also help stratify psoriasis patients according to molecular/immunological types. Such classification may be achieved through the delineation of distinct, biologically relevant modular A35 “sub-signatures.” For instance, our previous work identified distinct modular interferon signatures that formed the basis of a stratification system for SLE patients (20). Psoriasis classification might also be informed by measuring the changes in the abundance of other aggregates/modules. For instance, we observed changes in the abundance of transcripts comprising the A33 and A28 modules, (also associated with inflammation and interferon responses, respectively) in either one of the two psoriasis datasets. This was the case for A28 (interferon) in the GSE123787 fingerprints (**Figure 1**), which is in line with the interpretation contributed earlier by Catapano et al. (18).

Some of our findings may also be relevant from a drug discovery/repurposing standpoint. For one, a number of the gene products comprising the A35 signature are targeted by drug candidates for psoriasis treatment (**Table 2**). This finding suggests that—according to the principle of “guilt by association”—other valuable targets might be identified among the genes constituting these modules. Secondly, similarities observed between the psoriasis and Kawasaki disease fingerprints suggest that the pathogenesis and/or pathophysiology of these diseases might be driven, at least in part, by similar immune mechanisms. Kawasaki disease, also known as mucocutaneous lymph node syndrome, is a rare childhood disease that mostly affects children <5 years old (38, 40). This disease presents as an acute, self-limiting vasculitis that sometimes targets coronary arteries and causes ischemic heart disease (41–43). The parallels we drew between psoriasis and Kawasaki disease blood transcriptional signatures are consistent with the growing body of evidence showing that patients with Kawasaki disease can develop psoriasiform eruptions (44–48). Among the treatments approved for psoriasis, drugs inhibiting IL17 might be considered good candidates for repurposing in Kawasaki disease (9, 49). Independent reports have also associated Th17 responses (defined by IL17 production) with Kawasaki disease (50–53), and IL17 is a known driver of neutrophil development, recruitment, and activation (54, 55). We therefore posit that IL17 might constitute one of the factors underpinning the A35 signature that we identified here in patients with psoriasis and Kawasaki disease.

Several aspects of the benchmarking exercise that we have conducted here across independent studies are inherently limiting and need to be addressed in follow-on investigations. First, while the use of respective healthy control groups as common denominators permits comparisons across independent studies, further investigations should comprise cohorts of patients with psoriasis, Kawasaki disease, and healthy controls. Samples should be collected and processed using harmonized protocols and the generated data should be analyzed concomitantly using the same platform (RNA-seq). This approach would permit direct comparisons of psoriasis and Kawasaki disease profiles while minimizing technical sources of variation. Inclusion of healthy controls would help with the interpretation of the data and would also permit future data re-use and meta-analyses across independent studies.

Second, the cohort size should be sufficient to allow for investigations into inter-individual variability. Such investigations would permit, for instance, the identification of “endotypes” or distinct molecular phenotypes within each patient population. The relatively low cost of recently introduced RNA-seq protocols might help realize such sample sizes (e.g., QuantSeq 3’ mRNA-Seq by Lexogen: <$100/sample). Consolidating the results from multiple studies would remain feasible but any level of coordination or consultation between groups/centers could prove helpful.

Third, future studies are needed to clarify whether the A33/A35 signature observed in patients with psoriasis or Kawasaki disease is due to neutrophil priming secondary to inflammation or is a causal component of psoriasis pathophysiology. It can be difficult to ascertain in patient-based studies whether signatures are merely associated with or drive pathogenesis. Monitoring changes in transcript abundance at a high temporal frequency, either prior to a worsening of the clinical course of the disease or in response to therapy might provide useful indications in that sense. From a practical perspective, such studies could be implemented using protocols for at-home self-collection of low blood volumes and RNA stabilization (56, 57).

Finally, investigations into immune changes in the periphery and in bulk whole blood samples have inherent limitations. While systemic inflammation and interferon responses can be measured in whole blood, a dissection of the immune response at a more granular level (e.g., cellular subsets) might not be possible. Indeed, it is possible that we did not identify some responses associated with psoriasis pathogenesis (e.g., T-cell responses) for this reason. In addition, at least some immune responses may only be observed in affected skin tissues. Studies harnessing single-cell RNA-seq in a subset of patients are now warranted to further interrogate and interpret the psoriasis immune signatures measured in the peripheral blood.

Overall, our study provides a proof-of-principle for the use of fixed transcriptional module repertoires for blood transcriptome signature “benchmarking” and cross-study comparisons. It highlights the pertinence of using transcriptomic approaches for monitoring systemic inflammation. And it may also provide the necessary justification for further blood transcriptome studies in the context of psoriasis and Kawasaki disease.

## Data Availability Statement

Publicly available datasets were analyzed in this study. This data can be found here: https://www.ncbi.nlm.nih.gov/geo/ (the NCBI Gene Expression Omnibus).

## Author Contributions

AR, and DC: conceptualization. AR, KS and DC: data curation and validation. AR and DR: visualization. AR, AM, TK, MK, and ZT-C: analysis and interpretation. AR, DR, MT, MG, BK, DB, and NK: methodology development. AR and DC: writing of the first draft. AR, DR, MT, AM, TK, MG, ZT-C, NK, DB, MK, KS, and DC: writing—review and editing. The contributor’s roles listed above follow the Contributor Roles Taxonomy (CRediT) managed by The Consortia Advancing Standards in Research Administration Information (CASRAI) (https://casrai.org/credit/). All authors contributed to the article and approved the submitted version.

## Conflict of Interest

The authors declare that the research was conducted in the absence of any commercial or financial relationships that could be construed as a potential conflict of interest.

## Supplementary Material

Supplementary Figure 1 Module annotation grid. Complete annotation for the grid used to map the differences in transcript abundance between the cases and controls shown in **Figure 1**. Each position on the grid corresponds to a different module. Each of the 382 modules are constituted by a set of transcripts found to be co-expressed across a range of disease and physiological states. In turn, the modules are arranged in rows based on similarities in gene expression. Gene ontology, pathway or literature keyword enrichment analyses provide the basis for attribution of biological functions to the modules; these are indicated by the color-coded abbreviations list below the grid. An interactive presentation is available that permits the exploration of functional enrichment results and expression patterns for the A35 modules: https://prezi.com/view/7Q20FyW6Hrs5NjMaTUyW/.

## References

[1] Catapano M Vergnano M Romano M Mahil SK Choon S-E Burden DA Interleukin-36 promotes systemic Type-I IFN responses in severe psoriasis. bioRxiv (2018). p. 496851. 10.1101/496851

[2] Mousa A Misso M Teede H Scragg R de Courten B Effect of vitamin D supplementation on inflammation: protocol for a systematic review. BMJ Open (2016) 6(4):e010804. 10.1136/bmjopen-2015-010804 PMC4823456 27048637

[3] Paparella D Yau TM Young E Cardiopulmonary bypass induced inflammation: pathophysiology and treatment. An update. Eur J Cardiothorac Surg (2002) 21(2):232–44. 10.1016/S1010-7940(01)01099-5 11825729

[4] Michalek I Loring B John S A systematic review of worldwide epidemiology of psoriasis. J Eur Acad Dermatol Venereol (2017) 31(2):205–12. 10.1111/jdv.13854 27573025

[5] Wang J Suarez-Farinas M Estrada Y Parker ML Greenlees L Stephens G Identification of unique proteomic signatures in allergic and non-allergic skin disease. Clin Exp Allergy (2017) 47(11):1456–67. 10.1111/cea.12979 28703865

[6] Fry L Baker BS Powles AV Engstrand L Psoriasis is not an autoimmune disease? Exp Dermatol (2015) 24(4):241–4. 10.1111/exd.12572 25348334

[7] Meyer O Interferons and autoimmune disorders. Joint Bone Spine (2009) 76(5):464–73. 10.1016/j.jbspin.2009.03.012 19773191

[8] Hawkes JE Chan TC Krueger JG Psoriasis pathogenesis and the development of novel targeted immune therapies. J Allergy Clin Immunol (2017) 140(3):645–53. 10.1016/j.jaci.2017.07.004 PMC5600287 28887948

[9] Silfvast-Kaiser A Paek SY Menter A Anti-IL17 therapies for psoriasis. Expert Opin Biol Ther (2019) 19(1):45–54. 10.1080/14712598.2019.1555235 30500317

[10] van der Fits L Mourits S Voerman JS Kant M Boon L Laman JD Imiquimod-induced psoriasis-like skin inflammation in mice is mediated via the IL-23/IL-17 axis. J Immunol (2009) 182(9):5836–45. 10.4049/jimmunol.0802999 19380832

[11] Garber K Anti-IL-17 mAbs herald new options in psoriasis. Nat Biotechnol (2012) 30(6):475–7. 10.1038/nbt0612-475 22678368

[12] Conrad C Gilliet M Psoriasis: from Pathogenesis to Targeted Therapies. Clin Rev Allergy Immunol (2018) 54(1):102–13. 10.1007/s12016-018-8668-1 29349534

[13] Fyhrquist N Muirhead G Prast-Nielsen S Jeanmougin M Olah P Skoog T Microbe-host interplay in atopic dermatitis and psoriasis. Nat Commun (2019) 10(1):4703. 10.1038/s41467-019-12253-y 31619666 PMC6795799

[14] Tomalin LE Russell CB Garcet S Ewald DA Klekotka P Nirula A Short-term transcriptional response to IL-17 receptor-A antagonism in the treatment of psoriasis. J Allergy Clin Immunol (2020) 145(3):922–32. 10.1016/j.jaci.2019.10.041 31883845

[15] Brodmerkel C Li K Garcet S Hayden K Chiricozzi A Novitskaya I Modulation of inflammatory gene transcripts in psoriasis vulgaris: Differences between ustekinumab and etanercept. J Allergy Clin Immunol (2019) 143(5):1965–9. 10.1016/j.jaci.2019.01.017 30703387

[16] Li B Tsoi LC Swindell WR Gudjonsson JE Tejasvi T Johnston A Transcriptome analysis of psoriasis in a large case-control sample: RNA-seq provides insights into disease mechanisms. J Invest Dermatol (2014) 134(7):1828–38. 10.1038/jid.2014.28 PMC4057954 24441097

[17] Correa da Rosa J Kim J Tian S Tomalin LE Krueger JG Suarez-Farinas M Shrinking the Psoriasis Assessment Gap: Early Gene-Expression Profiling Accurately Predicts Response to Long-Term Treatment. J Invest Dermatol (2017) 137(2):305–12. 10.1016/j.jid.2016.09.015 27667537

[18] Catapano M Vergnano M Romano M Mahil SK Choon SE Burden AD IL-36 Promotes Systemic IFN-I Responses in Severe Forms of Psoriasis. J Invest Dermatol (2020) 140(4):816–26.e3. 10.1016/j.jid.2019.08.444 10.1016/j.jid.2019.08.444 PMC7097848 31539532

[19] Wang CQF Suarez-Farinas M Nograles KE Mimoso CA Shrom D Dow ER IL-17 induces inflammation-associated gene products in blood monocytes, and treatment with ixekizumab reduces their expression in psoriasis patient blood. J Invest Dermatol (2014) 134(12):2990–3. 10.1038/jid.2014.268 24999591

[20] Altman MC Rinchai D Baldwin N Toufiq M Whalen E Garand M Development and Characterization of a Fixed Repertoire of Blood Transcriptome Modules Based on Co-expression Patterns Across Immunological States. bioRxiv (2020). p. 525709. 10.1101/525709

[21] Barrett T Wilhite SE Ledoux P Evangelista C Kim IF Tomashevsky M NCBI GEO: archive for functional genomics data sets–update. Nucleic Acids Res (2013) 41(Database issue):D991–5. 10.1093/nar/gks1193 PMC3531084 23193258

[22] Novershtern N Subramanian A Lawton LN Mak RH Haining WN McConkey ME Densely interconnected transcriptional circuits control cell states in human hematopoiesis. Cell (2011) 144(2):296–309. 10.1016/j.cell.2011.01.004 21241896 PMC3049864

[23] Linsley PS Speake C Whalen E Chaussabel D Copy number loss of the interferon gene cluster in melanomas is linked to reduced T cell infiltrate and poor patient prognosis. PloS One (2014) 9(10):e109760. 10.1371/journal.pone.0109760 25314013 PMC4196925

[24] Wu Z Irizarry RA Gentleman R Martinez-Murillo F Spencer F A model-based background adjustment for oligonucleotide expression arrays. J Am Stat Assoc (2004) 99(468):909–17. 10.1198/016214504000000683

[25] Anders S Pyl PT Huber W HTSeq–a Python framework to work with high-throughput sequencing data. Bioinformatics (2015) 31(2):166–9. 10.1093/bioinformatics/btu638 PMC4287950 25260700

[26] Rinchai D Roelands J Hendrickx W Altman MC Bedognetti D Chaussabel D Blood transcriptional module repertoire analysis and visualization using R. bioRxiv (2020). 10.1101/2020.07.16.205963 PMC8388021 33624743

[27] Carvalho-Silva D Pierleoni A Pignatelli M Ong C Fumis L Karamanis N Open Targets Platform: new developments and updates two years on. Nucleic Acids Res (2019) 47(D1):D1056–65. 10.1093/nar/gky1133 PMC6324073 30462303

[28] Chaussabel D Quinn C Shen J Patel P Glaser C Baldwin N A modular analysis framework for blood genomics studies: application to systemic lupus erythematosus. Immunity (2008) 29(1):150–64. 10.1016/j.immuni.2008.05.012 PMC2727981 18631455

[29] Khaenam P Rinchai D Altman MC Chiche L Buddhisa S Kewcharoenwong C A transcriptomic reporter assay employing neutrophils to measure immunogenic activity of septic patients’ plasma. J Transl Med (2014) 12:65. 10.1186/1479-5876-12-65 24612859 PMC4007645

[30] Strober BE Gottlieb AB van de Kerkhof PCM Puig L Bachelez H Chouela E Benefit-risk profile of tofacitinib in patients with moderate-to-severe chronic plaque psoriasis: pooled analysis across six clinical trials. Br J Dermatol (2019) 180(1):67–75. 10.1111/bjd.17149 30188571 PMC7379291

[31] Menter MA Papp KA Cather J Leonardi C Pariser DM Krueger JG Efficacy of Tofacitinib for the Treatment of Moderate-to-Severe Chronic Plaque Psoriasis in Patient Subgroups from Two Randomised Phase 3 Trials. J Drugs Dermatol (2016) 15(5):568–80. 27168266

[32] He X Koenen H Smeets RL Keijsers R van Rijssen E Koerber A Targeting PKC in human T cells using sotrastaurin (AEB071) preserves regulatory T cells and prevents IL-17 production. J Invest Dermatol (2014) 134(4):975–83. 10.1038/jid.2013.459 24192715

[33] Wagner J von Matt P Faller B Cooke NG Albert R Sedrani R Structure-activity relationship and pharmacokinetic studies of sotrastaurin (AEB071), a promising novel medicine for prevention of graft rejection and treatment of psoriasis. J Med Chem (2011) 54(17):6028–39. 10.1021/jm200469u 21797275

[34] Chiang CC Cheng WJ Korinek M Lin CY Hwang TL Neutrophils in Psoriasis. Front Immunol (2019) 10:2376. 10.3389/fimmu.2019.02376 31649677 PMC6794444

[35] Schon MP Broekaert SMC Erpenbeck L Sexy again: the renaissance of neutrophils in psoriasis. Exp Dermatol (2017) 26(4):305–11. 10.1111/exd.13067 27194625

[36] Masson W Lobo M Molinero G Psoriasis and Cardiovascular Risk: A Comprehensive Review. Adv Ther (2020) 37(5):2017–33. 10.1007/s12325-020-01346-6 PMC7467489 32314303

[37] Hu SC Lan CE Psoriasis and Cardiovascular Comorbidities: Focusing on Severe Vascular Events, Cardiovascular Risk Factors and Implications for Treatment. Int J Mol Sci (2017) 18(10):2211. 10.3390/ijms18102211 PMC5666891 29065479

[38] McCrindle BW Rowley AH Newburger JW Burns JC Bolger AF Gewitz M Diagnosis, Treatment, and Long-Term Management of Kawasaki Disease: A Scientific Statement for Health Professionals From the American Heart Association. Circulation (2017) 135(17):e927–99. 10.1161/CIR.0000000000000484 28356445

[39] Kim J Tomalin L Lee J Fitz LJ Berstein G Correa-da Rosa J Reduction of Inflammatory and Cardiovascular Proteins in the Blood of Patients with Psoriasis: Differential Responses between Tofacitinib and Etanercept after 4 Weeks of Treatment. J Invest Dermatol (2018) 138(2):273–81. 10.1016/j.jid.2017.08.040 28927890

[40] Lin MT Wu MH The global epidemiology of Kawasaki disease: Review and future perspectives. Glob Cardiol Sci Pract (2017) 2017(3):e201720. 10.21542/gcsp.2017.20 29564341 PMC5856963

[41] Newburger JW Takahashi M Gerber MA Gewitz MH Tani LY Burns JC Diagnosis, treatment, and long-term management of Kawasaki disease: a statement for health professionals from the Committee on Rheumatic Fever, Endocarditis, and Kawasaki Disease, Council on Cardiovascular Disease in the Young, American Heart Association. Pediatrics (2004) 114(6):1708–33. 10.1542/peds.2004-2182 15574639

[42] Burns JC Glode MP Kawasaki syndrome. Lancet (2004) 364(9433):533–44. 10.1016/S0140-6736(04)16814-1 15302199

[43] de La Harpe M di Bernardo S Hofer M Sekarski N Thirty Years of Kawasaki Disease: A Single-Center Study at the University Hospital of Lausanne. Front Pediatr (2019) 7:11. 10.3389/fped.2019.00011 30761279 PMC6363689

[44] Sillen H Maes M Boiy T Wojciechowski M Plaque psoriasis following Kawasaki disease and varicella. BMJ Case Rep (2018) 2018:bcr-2018-224539. 10.1136/bcr-2018-224539 PMC6078230 30077967

[45] Ergin S Karaduman A Demirkaya E Bakkaloglu A Ozkaya O Plaque psoriasis induced after Kawasaki disease. Turk J Pediatr (2009) 51(4):375–7. 19950847

[46] Yoon SY Oh ST Lee JY Cho BK A plaque type psoriasiform eruption following Kawasaki disease. Pediatr Dermatol (2007) 24(1):96–8. 10.1111/j.1525-1470.2007.00349.x 17300666

[47] Mizuno Y Suga Y Muramatsu S Hasegawa T Ogawa H Psoriasiform and palmoplanter pustular lesions induced after Kawasaki disease. Int J Dermatol (2006) 45(9):1080–2. 10.1111/j.1365-4632.2005.02524.x 16961515

[48] Menni S Gualandri L Boccardi D Agostoni C Sala M Riva E Association of psoriasis-like eruption and Kawasaki disease. J Dermatol (2006) 33(8):571–3. 10.1111/j.1346-8138.2006.00134.x 16923141

[49] Toussi A Maverakis N Le ST Sarkar S Raychaudhuri SK Raychaudhuri SP Updated therapies for the management of psoriatic arthritis. Clin Immunol (2020) 108536. 10.1016/j.clim.2020.108536 32681979

[50] Jia S Li C Wang G Yang J Zu Y The T helper type 17/regulatory T cell imbalance in patients with acute Kawasaki disease. Clin Exp Immunol (2010) 162(1):131–7. 10.1111/j.1365-2249.2010.04236.x PMC2990938 20718783

[51] Guo MM Tseng WN Ko CH Pan HM Hsieh KS Kuo HC Th17- and Treg-related cytokine and mRNA expression are associated with acute and resolving Kawasaki disease. Allergy (2015) 70(3):310–8. 10.1111/all.12558 25585854

[52] Fitch E Harper E Skorcheva I Kurtz SE Blauvelt A Pathophysiology of psoriasis: recent advances on IL-23 and Th17 cytokines. Curr Rheumatol Rep (2007) 9(6):461–7. 10.1007/s11926-007-0075-1 PMC2893221 18177599

[53] Marinoni B Ceribelli A Massarotti MS Selmi C The Th17 axis in psoriatic disease: pathogenetic and therapeutic implications. Auto Immun Highlights (2014) 5(1):9–19. 10.1007/s13317-013-0057-4 26000152 PMC4389010

[54] Griffin GK Newton G Tarrio ML Bu DX Maganto-Garcia E Azcutia V IL-17 and TNF-alpha sustain neutrophil recruitment during inflammation through synergistic effects on endothelial activation. J Immunol (2012) 188(12):6287–99. 10.4049/jimmunol.1200385 PMC3370121 22566565

[55] Flannigan KL Ngo VL Geem D Harusato A Hirota SA Parkos CA IL-17A-mediated neutrophil recruitment limits expansion of segmented filamentous bacteria. Mucosal Immunol (2017) 10(3):673–84. 10.1038/mi.2016.80 PMC5350071 27624780

[56] Speake C Whalen E Gersuk VH Chaussabel D Odegard JM Greenbaum CJ Longitudinal monitoring of gene expression in ultra-low-volume blood samples self-collected at home. Clin Exp Immunol (2017) 188(2):226–33. 10.1111/cei.12916 PMC5383441 28009047

[57] Rinchai D Anguiano E Nguyen P Chaussabel D Finger stick blood collection for gene expression profiling and storage of tempus blood RNA tubes. F1000Res (2016) 5:1385. 10.12688/f1000research.8841.1 28357036 PMC5357033

