# Supplementary figures and images for: A Neutrophil-Driven Inflammatory Signature Characterizes the Blood Transcriptome Fingerprint of Psoriasis
Source: Front Immunol. 2020 Nov 24;11:587946. doi: 10.3389/fimmu.2020.587946 (PMC7732684; doi:10.3389/fimmu.2020.587946)

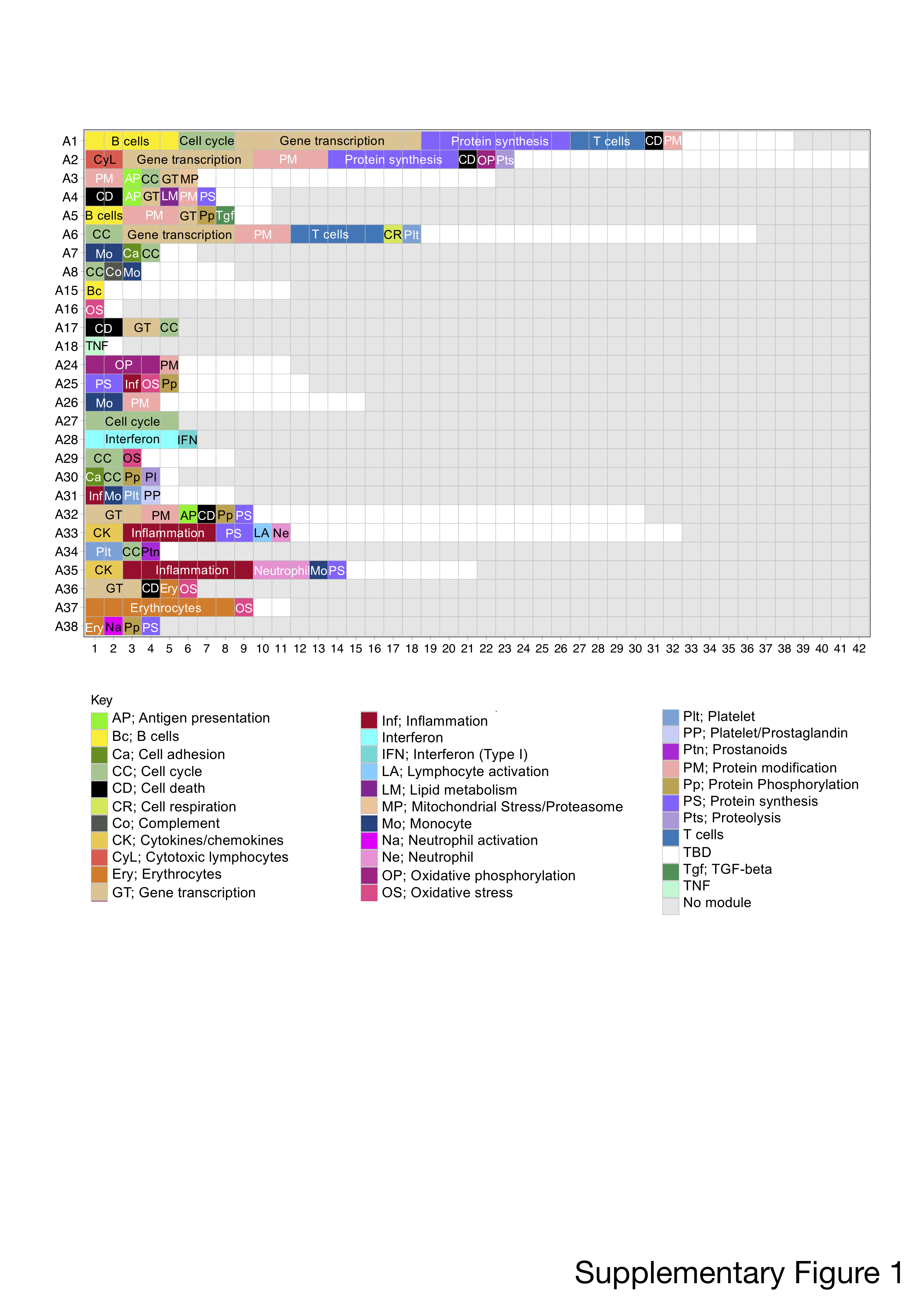

Supplement: Supplementary Figure 1 — Module annotation grid. Complete annotation for the grid used to map the differences in transcript abundance between the cases and controls shown in Figure 1 . Each position on the grid corresponds to a different module. Each of the 382 modules are constituted by a set of transcripts found to be co-expressed across a range of disease and physiological states. In turn, the modules are arranged in rows based on similarities in gene expression. Gene ontology, pathway or literature keyword enrichment analyses provide the basis for attribution of biological functions to the modules; these are indicated by the color-coded abbreviations list below the grid. An interactive presentation is available that permits the exploration of functional enrichment results and expression patterns for the A35 modules: https://prezi.com/view/7Q20FyW6Hrs5NjMaTUyW/. [file Image_1.jpeg]
